# Arabidopsis aneuploidy-like mutant *flpl1*, possesses enormous variations in multiple phenotypic characteristics

**DOI:** 10.1007/s10265-026-01712-5

**Published:** 2026-05-13

**Authors:** Asanga Deshappriya Nagalla, Kotaro Ishii, Tomonari Hirano, Sumie Ohbu, Yuki Shirakawa, Yusuke Kazama, Tomoko Abe

**Affiliations:** 1 Ion Beam Breeding Group, RIKEN Nishina Center for Accelerator-Based Science, Bioscience Building, 2-1 Hirosawa, Wako, Saitama 351-0198 Japan; 2 Department of Radiation Measurement and Dose Assessment, Institute for Radiological Science, National Institutes for Quantum Science and Technology, Chiba, 263-8555 Japan; 3 Faculty of Agriculture, University of Miyazaki, Miyazaki, Japan; 4 Department of Bioscience and Biotechnology, Fukui Prefectural University, Eiheiji-Cho, Japan

**Keywords:** Aneuploidy, Chromosome, Heavy-ion beam, Mutation

## Abstract

Heavy-ion beam irradiation is recognized as a powerful mutagenesis technique because it induces high mutation frequencies, causes minimal damage to other traits, and has the potential to produce large DNA-fragmentation mutants. Aneuploid cells may have an additional or fewer number of chromosomes compared to their wild-type. The mutant line of *flower late pltioleless1* (*flpl1*) is an ion beam-generated, *Arabidopsis thaliana* Col-0 based, aneuploid mutant line confirmed by whole genome sequence analysis, DNA quantity measurement using flow cytometry analysis and microscopic observations of an additional chromosome. The mutant shows abnormal leaf shapes, petioleless rosette leaves, elevated trichome bases and later-flowering phenotypes. Segregation analysis confirmed that the amount of additional DNA correlates with the observed phenotype as mutants with intermediate phenotypes possess moderate amounts of additional DNA. A set of candidate causal genes was identified using up and downregulated differentially expressed genes in transcriptome analysis. Gene ontology analysis further supports the mutant’s phenotypic characteristics and provides insights into its functional priorities, including the promotion of cellular structure and energy production. Meanwhile, the downregulation of senescence-related gene ontology terms validates its extended vegetative phase. The whole genome sequence-based chromosome rearrangement prediction and sequence coverage map analysis established the expected chromosomal model for *flpl1*. Ion beam mutagenesis generates aneuploidy and segmental aneuploidy mutants that may possess fused DNA-fragmented chromosomes.

**Supplementary Information:**

The online version contains supplementary material available at 10.1007/s10265-026-01712-5.

## Introduction

Mutations, the changes in the DNA sequence, are essential for genetic diversity, which may ultimately lay the foundation for natural selection. Mutations can arise from various sources, including natural processes, environmental factors, and human-induced methods such as chemical mutagenesis, e.g., Ethyl Methane Sulfonate (EMS) and radiation. γ-rays, X-rays and heavy-ion beams are the common sources of radiation mutagens. The heavy-ion irradiation causes dense ionization along the particle path, resulting in various degrees of DNA damage, and is recognized as an effective mutagenic tool for biological studies and product developments including plants and microbes (Hirano et al. 2013; Ichida et al. 2008; Ishii et al. 2024; Kanaya et al. 2008; Katano et al. 2016; Kazama et al. 2013a, 2016; Ma et al. 2013; Maeda et al. 2014; Nhat et al. 2021; Sasaki et al. 2012; Satoh and Oono 2019; Wang et al. 2013; Yamaguchi 2018; Yasui et al. 2012). In addition, heavy-ion irradiation possibly induces a wide range of mutations, including single nucleotide variants (SNVs), deletions and chromosomal level re-arrangements (Hirano et al. 2015; Kazama et al. 2012, 2017; Naito et al. 2005). The linear energy transfer (LET) of the heavy-ions is a crucial factor in determining the type and size of the mutation induced. The LETs available for biological experiments at the RI Beam Factory (RIBF) in RIKEN range from approximately 22.5–4,000 keV µm^−1^ (Ryuto et al. 2008). Higher-LET radiation deposits a high amount of energy that can damage the high-order structure of chromatin. It includes double-strand breaks (DSBs) that often cause errors in repairing (Hirano et al. 2012, 2013). Interestingly, the high LET can induce rare types and different sizes of mutations including large deletions, inversions, duplications and complex chromosomal rearrangements (Abe et al. 2021, 2025; Hase et al. 2017; Hirano et al. 2012, 2015; Ikoma et al. 2025; Kazama et al. 2011, 2013b, 2017; Morita et al. 2021; Oono et al. 2020; Sanjaya et al. 2021). It’s challenging to achieve these varieties of mutations with chemical mutagenesis agents such as EMS or colchicine.

Duplications and chromosomal rearrangements are types of chromosomal abnormalities that arise due to DSB followed by imprecise repair mechanisms. Ar-ion irradiation with 290 keVμm^−1^ was reported to induce a higher frequency of chromosomal-level rearrangements compared to other types of ion irradiation such as C-ions with 30 keVμm^−1^ (Hirano et al. 2012; Kazama et al. 2017). Mutant lines of Ar-365-as1 and Ar-443-as1 in the *Arabidopsis thaliana* (L.) Heynh. background were produced by Ar-ion irradiation, confirmed to have a 57.1 kbp duplicated region in Chr. 4 and a 2.26 Mbp, 318.1 kbp duplicated region in Chr 2, respectively (Hirano et al. 2015). Alone with duplications, Ar-57-al1, Ar-365-as1, and Ar-443-as1 mutants revealed a total of 22 DNA fragments that have contributed to complex chromosomal rearrangements. In addition to the Ar-ions, C-ions can also induce chromosomal rearrangements, even though their frequency is relatively low, at 10.2 for Ar-ions and 2.3 for C-ions rearrangements per mutant genome, respectively. The C-ion irradiated mutant lines C30-1-as1, C30-95-as1, and C30-108-as3 are reported to possess 5,4 and 3 rearrangement events, respectively (Kazama et al. 2017). Furthermore, multiple rearrangements occurred in localized regions of the chromosomes, indicating the heavy-ion beam can induce clustered DNA damage (Hirano et al. 2015). In addition to *A. thaliana*, a duplication event spanning about 2.5 kbp was reported in the rice Ne_50 mutant line induced by Ne ion irradiation with 31 keV μm^−1^ (Zheng et al. 2021).

DNA duplications and complex chromosome rearrangements may lead to serious chromosome abnormalities, including the existence of additional chromosomes, known as aneuploidy. The most common form of aneuploidy in humans is Down syndrome, which is caused by trisomy of Chr. 21 in patients, where it’s diploid in nature. Down syndrome patients show a set of unique types of characteristics, yet they depend on the genomic and environmental variabilities (Antonarakis et al. 2004). Even though there are several genes on Chr. 21 confirmed to be causal for Down syndrome, the complexity of gene dosage effects occurred not only due to the expression of genes on Chr. 21, but also global gene expression patterns across the genome (Letourneau et al. 2014). On the other hand, plants have proven to be more tolerant of aneuploidy mutations. All five possible trisomics of *A. thaliana* mutants are viable and have unique phenotypes (Rédei et al. 1992) and a highly heterogeneous aneuploidy population consisting of 25 different karyotypes has been previously reported (Henry et al. 2010). Those aneuploidy mutants exhibit a few key phenotypic characteristic differences compared to their euploid counterparts, yet the degree of severity of each phenotype is variable (Henry et al. 2010; Ramsey and Schemske 1998). However, an aneuploid *A. thaliana* mutant derived from heavy-ion beam irradiation may not be a classical one (mutants derived from chromosome doubling agents such as Colchicine) because the responsible chromosome would be a product of duplications, complex chromosomal rearrangements and various degrees of other mutations. An irradiation experiment conducted with *Parachlorella kessleri*, a unicellular green alga, resulted in complex, fragmented chromosomes. The wild-type *P. kessleri* possesses seven A chromosomes and three B chromosomes. After irradiation with 50 Gy Fe-ion beam and subculturing, the mutants contained more than 20 chromosomes, and the segmental chromosomes were shown to be heritable (Ishii et al. 2025).

In the current study, we identified a C-ion with a 30 keVμm^−1^ irradiated *A. thaliana* mutant that shows a series of phenotypic changes compared to wild type (WT). The pattern of inheritance of the mutant characters does not follow the Mendelian genetics ratios and flow cytometry analysis revealed that the mutant genome is significantly larger than the WT genome. The mutant cells were observed using a high-resolution microscope and an additional chromosome was observed. The whole genome sequencing (WGS) read depth has increased by 0.5-fold across the entire chromosome 2, confirming the mutant possesses an additional copy of chromosome 2. Due to the presence of an extra chromosome, the whole transcriptome of the mutant shows a misbehavior. The Gene Ontology (GO) analysis highlighted that the mutant is actively modifying processes related to structural components, enhancing metabolic activity while downregulating senescence-related processes. Our result provides evidence of the correlation between morphological abnormalities with DE genes and their GO terms triggered by the heterogeneous chromosomal fragments.

## Materials and methods

### Plant materials including F_2_ population, flowering date and phenotype observations

The *A*. *thaliana* accession Columbia-0 (Col-0) was used as the WT plant. The C30-81-as6 (*flpl1*) mutant line was derived from C-ion irradiation (400 Gy, 30 keVµm^−1^) on Col-0 dry seeds as the method described in Kazama et al. (2011). The mutant was identified in the M_2_ generation, and self-fertilized seeds from the best mutant (later flowering, crumpled, large and petiole-less rosette leaves: see the results section) named as *flower late pltioleless1* (*flpl1*) were selected and grown for the next generation, up to the M_7_ generation. The F_2_ population is derived by backcrossing with Col-0, and self-fertilization of the resulted F_1_ population. The *flpl1*-77 plant, which showed the strongest phenotype observed among the F_2_ population of 84 individuals, was selected. Seed preparation, germination, cultivation and transplanting were done based on the method described in Kazama et al. (2011). Flowering time was monitored from the days after germination, and growth room conditions were maintained constant throughout the experiment. The *flpl1* mutant used in this study were derived from the M_3_ generation unless it’s specified otherwise. To confirm the stability of the mutant trait, the best mutant was selected and grown up to the M_7_ generation.

### Whole-genome mutation analysis

A *flpl1* plant in the M_3_ generation and *flpl1*-77 plants were selected and the DNA was extracted from leaf samples using DNeasy plant mini kit (QIAGEN, Venlo, Netherlands), with using IDTE 1 × TE solution pH 8.0 (Integrated DNA technologies, Iowa, USA). The DNA libraries were created using MGIEasy PCR-Free DNA library prep kit (MGI Tech, Shenzhen, China). The whole genome sequencing was performed using MGI DNBSEQ-G400 instrument (MGI Tech) in paired-end, 2 × 150-bp mode. Bioinformatics analysis was conducted using AMAP (Ishii et al. 2016) implemented on the HOKUSAI-BigWaterfall supercomputing system (RIKEN). The AMAP includes programs for quality control, mapping, detection of various degrees of mutation and creating a final analysis integrating the analysis results. The resulting candidate mutations were visually confirmed using IGV software (Robinson et al. 2011).

### Microscopic observation of the chromosomes

The Chromosome observation was done using 0.4 mm diameter flower buds. The selected flower buds were fixed in the fixative solution (5:1(Methanol: 0.2 M Acetic Acid)) under vacuum in a 0.5 mL tube. After overnight fixation, the solution was replaced with 70% Methanol and kept overnight at 4 °C. Next, the buds were washed twice with distilled water. The moisture-removed buds were transferred to an enzyme solution (Macerozyme: 0.08 g, Pectolyase: 0.02 g, Cellulase: 0.1 g and dissolve in 10 mL of acetic acid buffer (pH 4.2)) and incubate for 50 min in 37 °C. The digested and moisture removed buds were placed on a glass slide. The buds were crushed with fine tweezers and spread using a fixative solution. 7 µL of DAPI (1 mg/mL) was added to the tissues and covered with a cover glass. The FV3000 Inverted Confocal Laser Scanning Microscope (Evident Corporation Tokyo, Japan) with 10 × 100 (100X/1.40 Oil UPlanSApo) magnification under a 405 nm laser was used for chromosome observation.

### Flow cytometry analysis

The nuclear ploidy level was observed by using a flow cytometer (CyFlow counter, Sysmex, Kobe, Japan). Fully mature leaf samples (1 cm^2^) of the same age (days after germination) Col-0 and *flpl1* mutants were collected and chopped with 200 μL of Otto I buffer (Otto 1990). Then the residue was filtered through a 30 µm nylon mesh filter to remove debris. The DNA staining solution (Mishiba et al. 2000) 800 µL was added to the filtrate and stained for 1 min. Peaks in flow cytometry analysis were detected using the flow cytometer’s default settings, and the average fluorescence intensity of the nuclei contained within the peaks was calculated.

### Transcriptome and GO enrichment analysis

Fully mature leaf samples from three individual plants of *flpl1* mutant and WT were collected. All the plants were the same age (days after germination) and *flpl1* mutants were confirmed as positive mutants by phenotypic observation and the presence of additional DNA content by flow cytometric analysis. The total RNA was extracted using the RNeasy Plant Mini Kit (QIAGEN, Venlo, Netherlands). The cDNA library preparation was conducted by using VAHTS Universal V8 RNA-seq Library Prep kit (Nanjing, PRC). An Illumina Novaseq 6000 instrument was used for sequencing and a 2 × 150 paired-end configuration was adopted according to manufacturer’s instructions. Quality control was conducted using Cutadapt (V1.9.1, phred cutoff: 20, error rate: 0.1, adapter overlap: 1 bp, min. length: 75, proportion of N: 0.1) (Martin 2011) to obtain high quality data. Clean reads of each biological sample were aligned to the *A. thaliana* genome assembly (TAIR10) by using Hisat2 (v2.2.1) (Kim et al. 2015). The HTSeq (v0.6.1) software package (Anders et al. 2015) was used to count the reads mapped to genomic features, and to estimate gene and isoform expression levels from the pair-end clean data. Fragments Per Kilobase of transcript per Million mapped reads (FPKM) plot box distribution indicated that median values remain relatively similar among all the Col-0 and mutant samples indicating that the central tendency of gene expression levels is comparable (Fig. S1). This suggested that the occurrence of the additional chromosome hasn’t created a global expression shift and DEG analysis can be performed without additional normalizations. The resulting count matrix was used for DEG analysis using DESeq2 (Love et al. 2014) Bioconductor package, a model based on the negative binomial distribution. The DE genes were identified by adjusted *p*- value < 0.05 and a minimum two-fold change in expression. The GO enrichment analysis was conducted considering DE upregulated and downregulated genes separately using the R package clusterProfiler (v4.0) (Yu et al. 2012) with *A. thaliana* annotations from org.At.tair.db (Huber et al. 2015). The Gene Ontology resource (Consortium 2021) was used as the reference database. Terms with FDR (q-value) < 0.05 are considered significant. The WGS coverage analysis was conducted using R packages including Rsamtools (v2.18.0) (Morgan et al. 2023), GenomicRanges (v1.54.1), and GenomicAlignments (v1.38.2) (Lawrence et al. 2013) for BAM file processing and genomic coordinate management. Copy number variation detection was performed using DNAcopy (v1.76.0) (Venkatraman and Olshen 2007).

## Results

### Detection of multiple phenotypic abnormalities in the *flpl1* mutant line

**Fig. 1:**
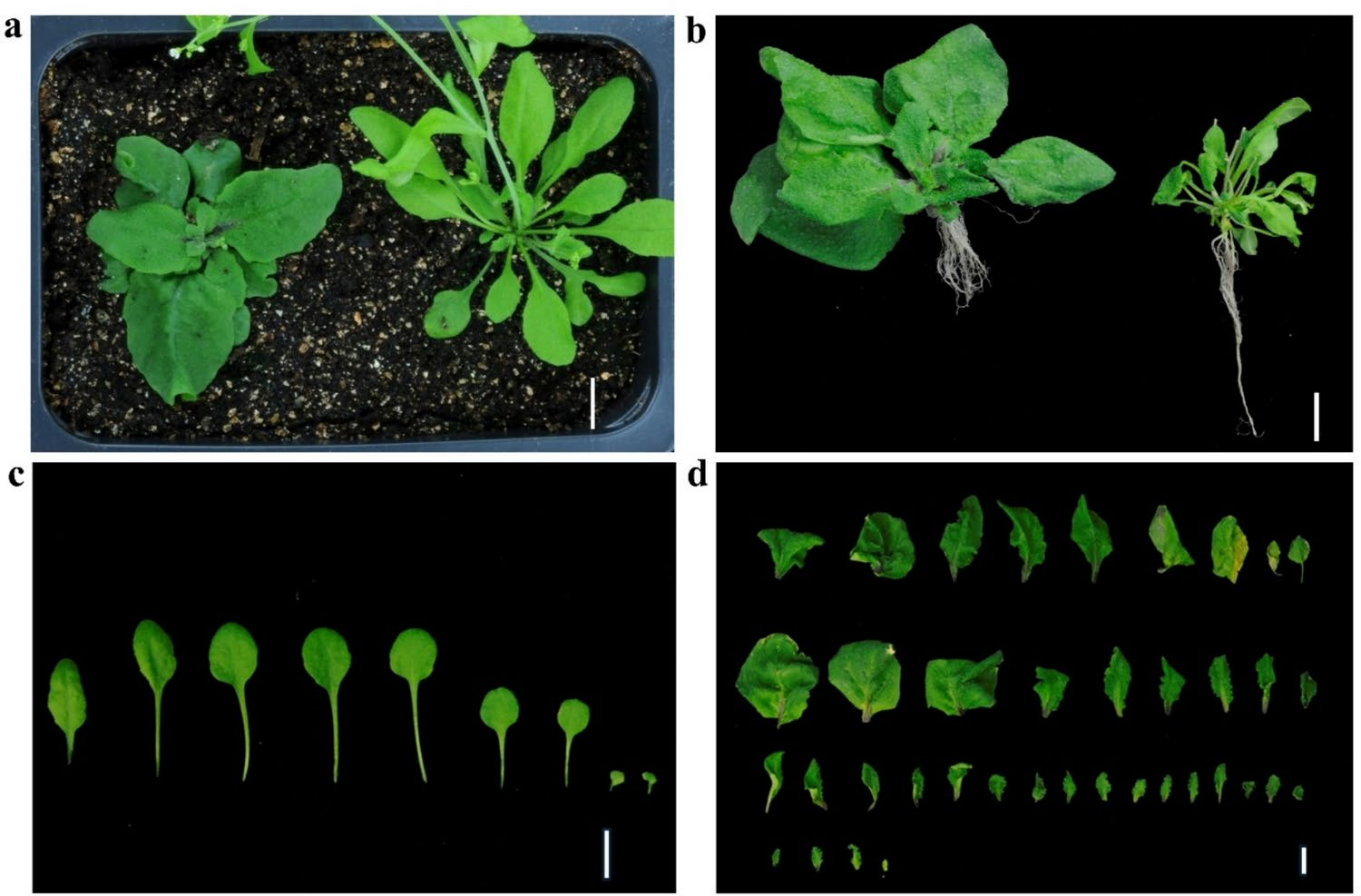
Phenotypic characteristics of the *flpl1* mutant and WT plants. **a** The mutant (left) flowered late, with large and petiole-less rosette leaves compared to the WT (right). **b** The mutant possesses a shallow root system (left) compared to the WT (right). The leaf panel of WT (**c**) and *flpl1* (**d**) shows the differences in leaf morphology and the number of leaves at the flowering stage. Photos (**a** and **b**) were taken when the *flpl1* showed flowering around 43 days after germination. Bars = 1 cm

The M_3_ plants of C30-81-as6, identified as a mutant line with abnormal leaf morphology, exhibited multiple phenotypic alterations, including delayed flowering, which occurred approximately six weeks after germination, in contrast to the wild-type plants that flowered after around two week (Table 1). Typical *flpl1* mutants had wrinkled, large leaves and rosette leaves without petioles (Fig. 1a, b, d). The mature leaves are large, wide, thick and fleshy with clear sinuate edges compared to WT mature leaves. This mutant is delayed in flowering and has an increased number of rosette leaves during the long vegetative period (Table 1 and Fig. 1d). We grew inbred populations up to the M_7_ generation to check the stability of mutant characters. The M_7_ mutants have the resulted resemble phenotypic features, including the delay of flowering time (Table 1).

**Table 1:** Flowering time (days) of the *flpl1* mutant and WT plants. Data are means ± SD (*n* ≥ 4)

Line	M_3_	M_7_
Col-0	15.1 ± 0.9	16.6 ± 1.0
*flpl1*	43 ± 2.8^*^	40.3 ± 5.6^*^

^*^The significance of the difference was assessed by Student’s *t*-test (*p < 0.01)

**Fig. 2:**
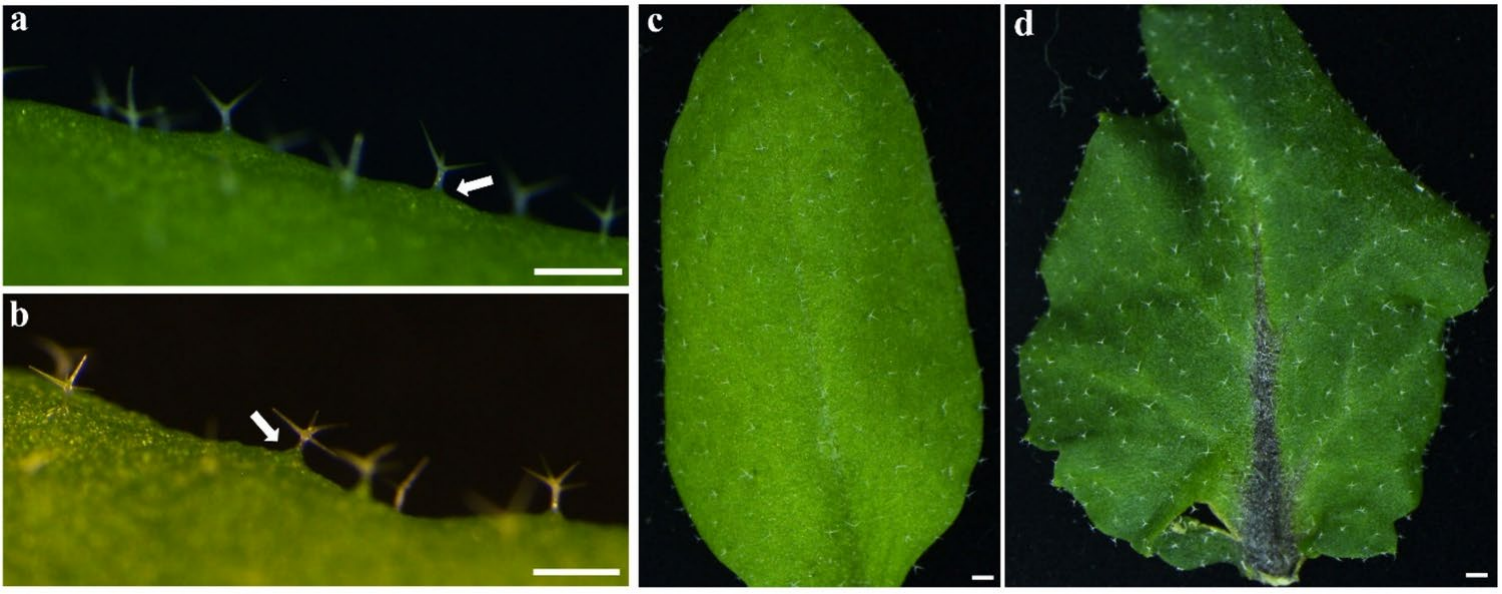
Trichome base and leaf base morphology of the *flpl1* mutant and WT plants. The trichome base of the Col-0 (**a**) is relatively flat but the mutant (**b**) has a heap-shaped trichome base. The white arrows point to the trichome base. The leaf surface of the Col-0 (**c**) and the mutant (**d**). Bars = 0.5 cm

The leaves of the *flpl1* appeared to have a rougher surface than those of the WT. Initially, we suspected that this was due to an increase in trichome density. However, microscopic observations revealed that there was no significant increase in trichome density (Table S1); instead, the trichome bases were elevated. The trichome bases of the mutant exhibited a mountain-like shape. In contrast, those of the WT appeared flat (Fig. 2). In addition to leaf morphology, the *flpl1* showed interesting root structural abnormalities compared to the WT. The mutant had a shallow and dense rhizosphere, whereas the WT had a relatively deep and thin rhizosphere (Fig. 1b).

### The ***flpl1*** does not follow the Mendelian genetic ratios of inheritance

There were various types of segregants in the M_3_ population. Approximately 3.3% exhibited all of the above phenotypic characteristics and were designated as *flpl1*. Mutants lacking any of these characteristics: later flowering, crumpled, large and petioleless rosette leaves were considered non-*flpl1*. To obtain a homozygous population, we grew several inbred populations. The *flpl1* mutant with strong phenotypes was selected and its seeds were used to grow the next population. The percentage of *flpl1* plants in total plants was 9.0%, 25.0% and 36.8% in the M_4_, M_5_ and M_7_ generations, respectively (Table S2). Therefore, the occurrence of the positive mutants was low in consecutive inbred populations, indicating the possibility of multiple genes responsible for the positive mutant phenotype.

### Investigation of the gene responsible for the phenotypic change in the *flpl1*

To investigate the responsible gene/s for the phenotypic variations in the *flpl1* mutant, first, we tried WGS analysis. The automated mutation analysis pipeline (AMAP; Ishii et al. 2016) resulted in six SNVs, one deletion, and 13 chromosomal breakpoints, including intra-chromosomal translocations (ITX), and inter-chromosomal translocations (CTX). Totally, nine homozygous mutated genes were found (Table S3). The mutations were confirmed by manual observation in Integrative Genomics Viewer (IGV; Robinson et al. 2011). Next, we performed a linkage analysis using an F_2_ population, but none of the nine homozygous mutated genes were genetically linked with at least a single mutant phenotype (Supplementary Text 1 and Fig. S5).

### Increased DNA content and chromosome numbers in the *flpl1*

**Fig. 3:**
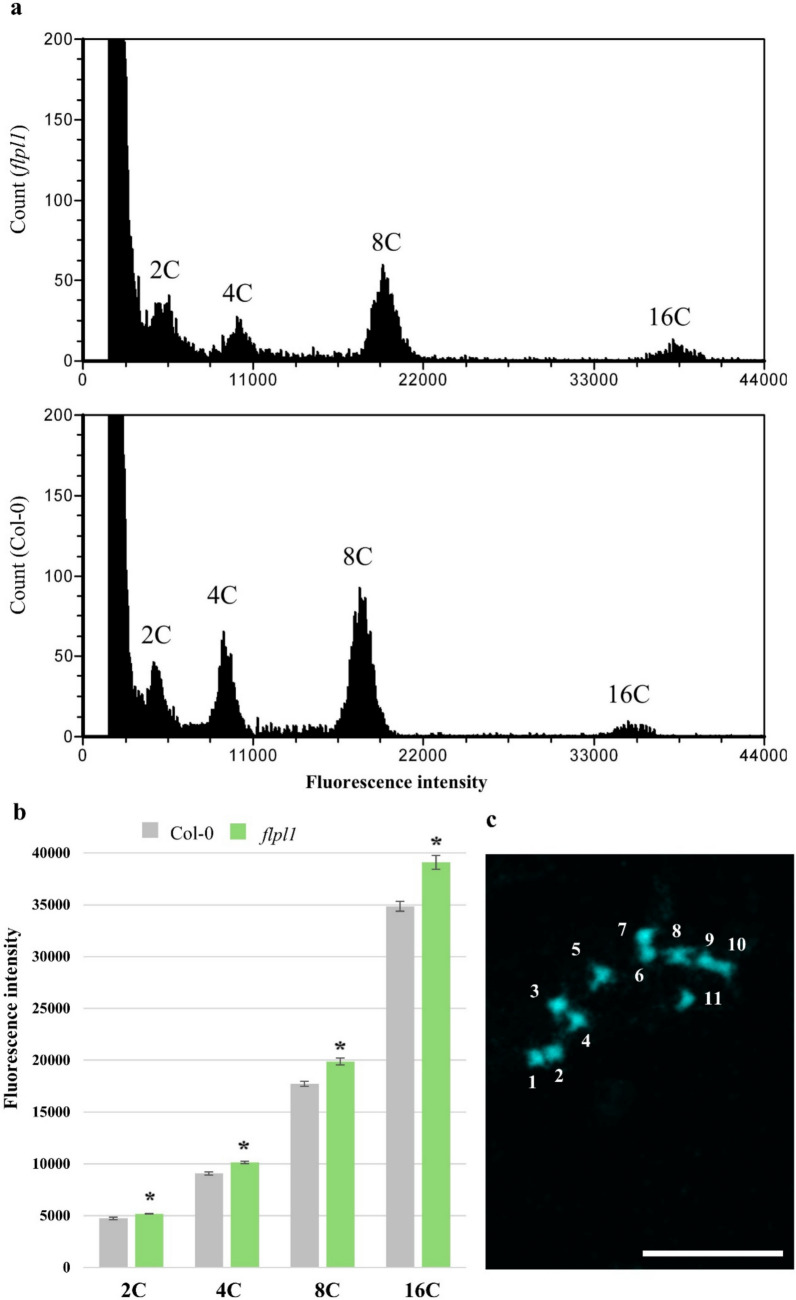
Flow cytometry analysis in *flpl1* and Col-0 plants. **a** Histogram of fluorescence intensity of the nuclei in mutant (upper) and Col-0 (below). 2C, 4C, 8C and 16C peaks represent the diploid, tetraploid, octoploid and hexaploid nuclei, respectively. **b** Comparison of the fluorescence intensity for each peak. The significance of the difference was assessed by Student’s *t*-test (*p < 0.01) and *n* ≥ 3. **c** Microscopic observation of chromosomes. Chromosome observation of the cells from flower bud samples of *flpl1* in M_3_ generations under 1,000× magnification. The numbers 1–11 represent the number of chromosomes (The chromosome numbers do not represent actual *A. thaliana* chromosomes). Bar = 10 μm. Chromosome observation was performed twice using flower buds from an independent generation and obtained the same results

We performed a flow cytometry analysis to measure the genome size of the mutant using leaf samples of Col-0 and *flpl1* plants. The histogram indicates four clear peaks of nuclear DNA content. The 2C peak representing diploid nuclei shows a slight shift in DNA content in the mutant. Importantly, the 4C, 8C and 16C peaks represent the tetraploid, octoploid and hexaploid nuclei showing a clear shifting of DNA content in the mutant compared to Col-0, suggesting significantly increased genomic DNA levels (Fig. 3). The ratio of DNA content of Col-0 and *flpl1* for each peak is 1.1 (2C:1.09, 4C:1.12, 8C:1.12, 16C:1.12). This data on the additional DNA content reflected the possibility of having an additional chromosome in the mutant genome.

To ascertain this possibility, we observed the cell nuclei of M_3_ and M_7_ (photo is not shown) mutants (additional DNA content was verified by flow cytometry analysis for each mutant plant) flower bud samples. A total of 11 chromosomes were observed in the candidate cells undergoing division (Fig. 3c).

**Fig. 4:**
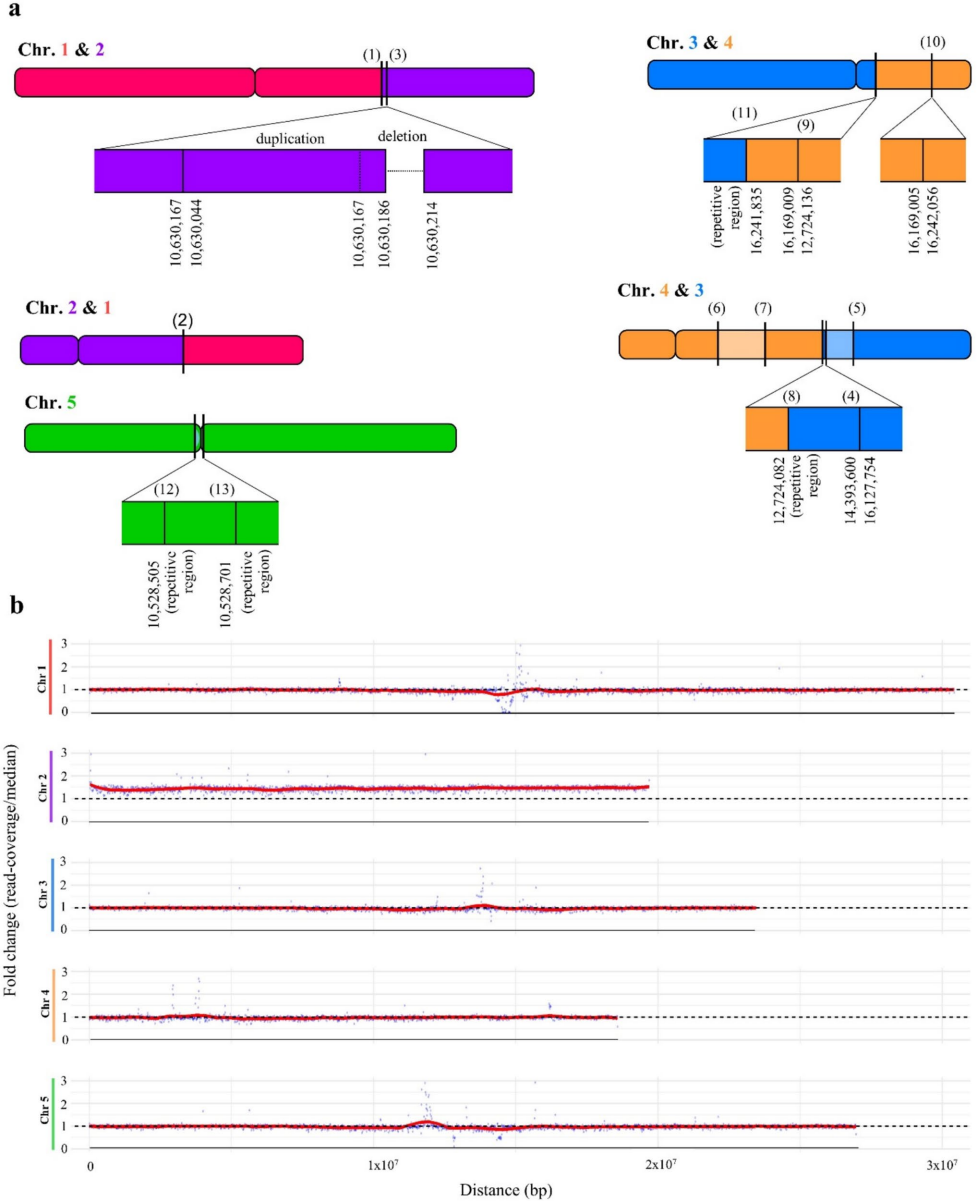
Visualization of additional chromosome fragments **a** diagram of proposed reconstructed chromosome structures of the *flpl1* mutant based on the Automated Mutation Analysis Pipeline. The mutation IDs (1–13) represent the candidate break\rejoin locations as indicated in Table S3, based on TAIR 10. The panel below the chromosomes shows a magnified regionof mutations with actual positions. Pale-colored fragments indicate those rejoined in an inverted direction and the sizes of the chromosome fragments are proportionate to the actual nucleotide sizes. **b** Whole genome sequence coverage plot for each chromosome. Coverage refers to the average reads per base in a 10 kbp window indicated in blue dots (bin size = 10 kbp) and the median refers to the middle coverage value across the entire genome. The red trend lines show the smoothed average coverage pattern across the genome and the black dashed line refers to the base reference line. The x-axis shows positions along the chromosome in base pairs

To elucidate potential chromosome rearrangements, fragment breakpoints and rejoined junctions were visualized using WGS data analysis. A reciprocal, CTX between Chr. 1 and Chr. 2 resulted in a tandem duplication and a deletion in the second repeat in Chr. 2 (mutation ID 1 and 2 in Fig. 4a). Since the Chr. 1 side (the side of the break-point) is homozygous and the Chr. 2 side is heterozygous (mutation ID 2), it is considered that there could be another copy of Chr. 2 that does not carry the translocation (Table S3). This assumption was verified by WGS coverage mapping across each of the chromosomes. A clear 50% increment of short-read sequences was mapped on the entire Chr. 2, confirming the presence of an additional copy of chromosome 2 (Figs. 4b, S2). Another candidate reciprocal CTX was reported between Chr. 3 and Chr. 4. However, the exact position on the Chr. 3 side is unknown due to the occurrence of the repetitive region (mutation ID 8). An intra-chromosomal translocation with an inversion was observed in Chr. 5, although the correct positions are unknown due to repetitive sequences (mutation ID 12 and 13). The *flpl1*-77 is the best mutant from the BC_1_F_2_ population showing an intermediate phenotype (Fig. S3) compared to *flpl1*. We performed WGS analysis and sequence coverage mapping using its genomic DNA. Interestingly, the *flpl1*-77 mutant contained an additional chromosome, which is made of fused fragments of Chr. 1 and Chr. 2 (Fig. S3) with a similar positioned CTX (as resulted in *flpl1*). Therefore, it can be identified as an additional copy of *flpl1*’s Chr. 1. Both sides of Chr. 1 and Chr. 2 were heterozygous, possibly due to the presence of native copies of chromosome 1 and 2, since the *flpl1*-77 has undergone an event of back-crossing (Table S4).

Therefore, both chromosomal rearrangement and WGS coverage mapping analysis propose the occurrence of an additional copy of Chr. 2. Since we were unable to confirm the genetic linkage with other homozygous mutated genes with the mutant phenotype, the presence of the CTX in Chr.1- Chr. 2 and the additional chromosome would be the key mutations responsible for *flpl1* phenotypic characters.

### Segregation analysis of mutant phenotypes in the *flpl1* line

**Fig. 5:**
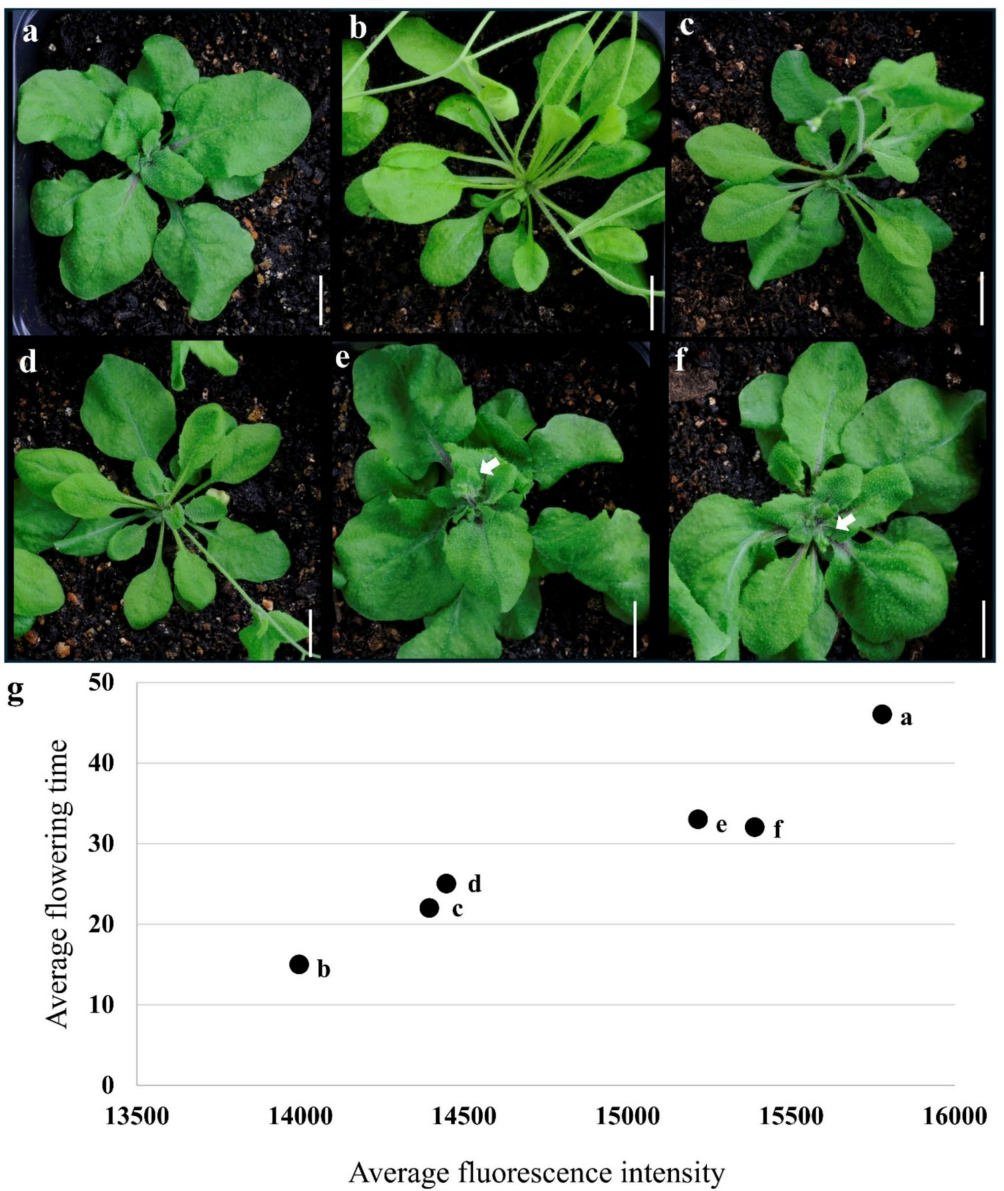
The plants with intermediate additional DNA content show weaker phenotype characters compared to *flpl1*. **a** *flpl1* mutant. **b** WT. **c**, **d** *flpl1* mutants show moderate mutant phenotypes in flowering time and leaf shape compared to WT. **e**, **f** *flpl1* mutants show strong mutant phenotypes in leaf shape and flowering time compared to WT. **g** Flowering time is delayed with an increase in fluorescence intensity in each mutant (**a**–**f**). Plants of the M_3_ segregating population were used and white arrows indicate the emergence of immature flower buds (around 32 days after germination). Bars = 1 cm

Throughout the M_3_-M_7_ generations, we found plants showing intermediate characteristics, including leaf shape and flowering time. Next, we were interested in understanding the correlation of additional DNA content in controlling mutant phenotypic characters. In a situation of partial aneuploidy where the additional chromosome is expected to be compaction and segmental duplications occur in meiosis, it’s possible to obtain plants with intermediate DNA content (DNA amount is greater than WT, but less than aneuploidy mutant: *flpl1*) (Henry et al. 2005, 2010). Therefore, we checked the DNA content of the intermediate phenotype display plants, mutants and WT using flow cytometry. As expected, the early flowering plants show weak leaf phenotypes (shape and size) and less additional DNA compared to *flpl1* mutants. When comparing the DNA content of several mutants that flower between the earliest-flowering wild type and the latest-flowering mutant, the increase in DNA content was greater in the later-flowering mutants (Fig. 5). These results suggest that the increased DNA content is associated with the multiple mutant phenotypes. The AMAP suggested the increased DNA content associated with mutant phenotypes belongs to an additional copy of Chr. 2. To assess this possibility, we performed a quantitative PCR (qPCR) analysis targeting six PCR amplicons on Chr. 2 (Fig. S4d and Table S7) using the same amount of *flpl1* and *flpl*-weak mutants’ DNA. These *flpl1*-weak mutants display almost similar phenotypes as WT except leaf shape-related abnormalities (Fig. S4b) since those plants may exhibit segmental duplications. The qPCR data resulted in a significant increment of relative DNA quantities in *flpl1* compared to WT in all the tested amplicon sites (Fig. S4e). In addition, the *flpl1* resulted in an almost 1.5-fold quantity increment compared to *flpl1*-weak2. These qPCR results confirm the increased DNA quantities in Chr. 2 would be the cause for the *flpl1* phenotype.

### The *flpl1* mutant possesses an extremely misregulated transcriptome compared with WT

**Fig. 6:**
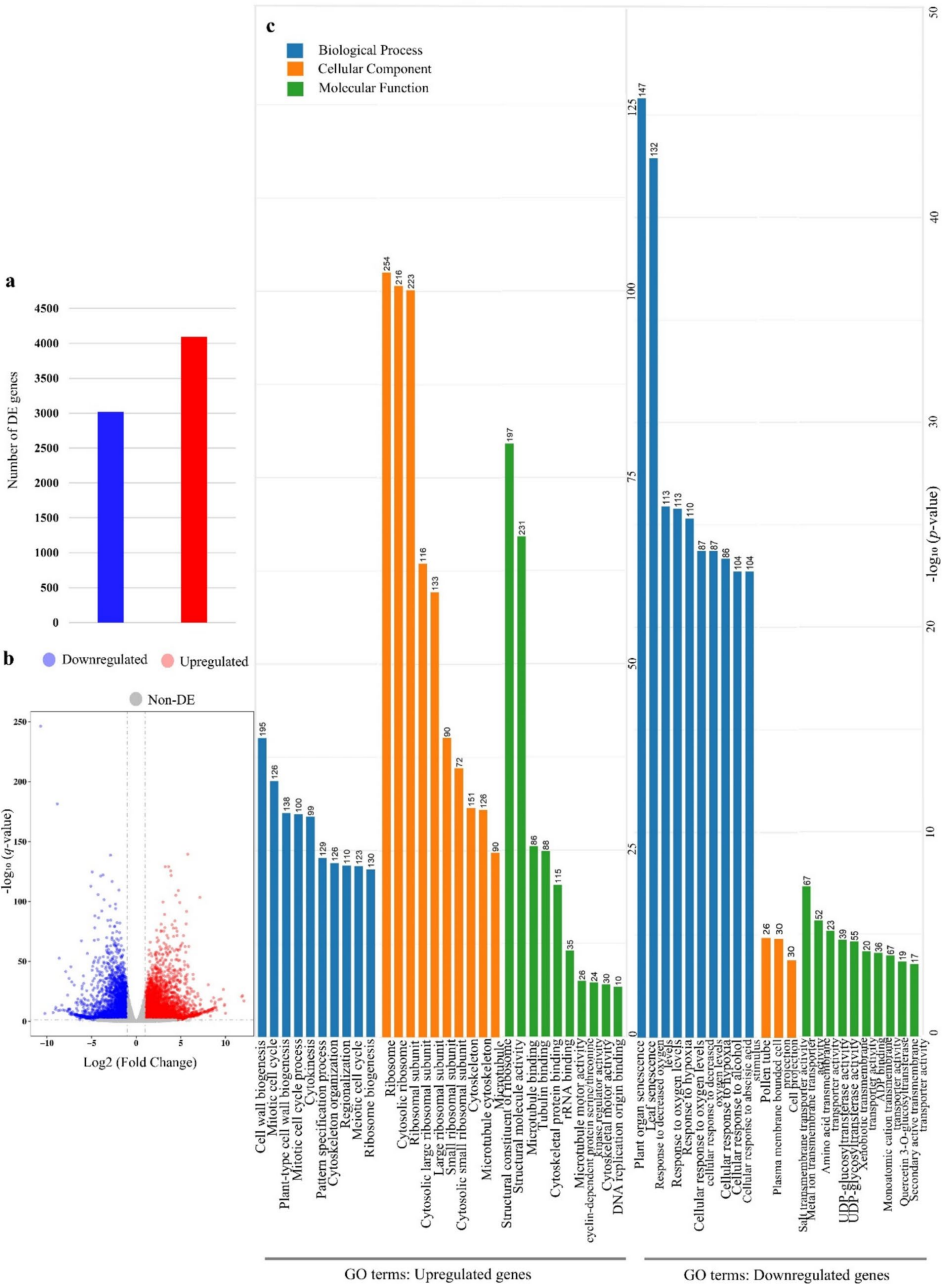
Overview of transcriptome analysis results of *flpl1* mutants and Col-0. **a** the number of up and downregulated DEGs in the mutant compared to Col-0. **b** volcano plot showing the distribution of significantly up and downregulated DEGs. **c** a summary of gene ontology (GO) terms for up and downregulated DE genes. The top 10 statistically significant GO terms show in the bar plot and the number in front of the bar represents the number of genes supported for the corresponding GO term

Based on the above results, the mutant shows a series of phenotypic deviations due to the segregation of an additional chromosome. Because of the appearance of phenotypic defects, we hypothesized that the mutant’s transcriptome would be severely misregulated, mainly due to the overexpression of a cluster of genes positioned on the duplicated regions. To understand the impact on the whole transcriptome, we performed a transcriptome analysis using the positive mutants confirmed by flow cytometry analysis and Col-0 plants. A total of 4,096 upregulated and 3,022 downregulated DEGs were identified (Fig. 6). The total number of DEGs accounted for about 30% of the whole transcriptome. The GO analysis for upregulated genes emphasized that the most significant and the highest number of genes are involved in plant-type cell wall biogenesis, mitotic cell cycle and ribosome biogenesis and as the key biological processes (BP), structural molecular activity, including structural constituents of ribosome and cytoskeleton protein binding as the key molecular functions (MF) and the Cellular Component (CC) of most genes are ribosome, ribosomal subunit, and cytosolic ribosome. The GO terms for downregulated genes resulted that, the highest number of genes with significance recorded in BP being plant organ senescence, leaf senescence and response to decreased oxygen levels. Salt transmembrane transporter activity, metal ion transmembrane transporter activity and UDP-Glycosyltransferase activity are results under the top three GO terms for MF. The downregulated DEGs, which are functionally connected to CC are plasma membrane-bounded cell projection, cell projection and pollen tubes (resulting in only three GO terms). However, the number of genes identified in CC is quite low compared to BP and MF (Fig. 6c). Collectively, the GO terms for upregulated genes outline the mutants’ prioritization for structural components production, energy and precursor molecule acquisition. Interestingly, the plant senescence regulatory genes are strictly downregulated in the mutant, providing the genetic basis for the extended vegetative phase supported with structural components produced by upregulated genes.

### Transcriptome analysis provided insight into the mutant phenotype

An additional chromosome is expected to be responsible for increasing the number of copies of a gene and possibly the gene may be overexpressed. However, based on the transcriptome data, both upregulated and downregulated DEGs resulted (Fig. 6a, b). This may be due to the feedback transcriptional regulation, indicating that the overexpressed and downregulated genes are responsible for the opposite biological function. The number of 1.5-fold upregulated (*p*adj- < 0.05) genes on Chr. 2 (1,378) was slightly greater than in Chr. 1 (1,290) and Chr. 5, resulting in 1,133 upregulated genes. This result shows that simply upregulated genes in Chr. 2 do not cause the *flpl1* phenotype, rather, the instability of the global transcriptome would be the cause. Based on the previous reports, the aneuploidy may disrupt the expression of many genes on triplicated chromosomes as well as non-triplicated chromosomes (Huettel et al. 2008).

Transcriptome data provide clues to explain the mutant’s abnormal phenotypic features. The *flpl1* is a flowering time-related mutant and both downregulation of important floral promoters and upregulation of floral repressors were reported. The floral promoters include *FLOWERING LOCUS T* (*FT*), *GIGANTEA*, *Flavin-Binding -Kelch Repeat-F-Box 1* (*FKF1*) and *FRUITFULL* (*AGL8*), which were downregulated in the mutant. Meanwhile, upregulation of floral repressors *TERMINAL FLOWER 1* (*TFL1*), *CURLY LEAF* (*CLF*), *VERNALIZATION 5* (*VRN5*) and *AGAMOUS-LIKE 15* (*AGL15*) were observed in the mutant. Simultaneously, *flpl1* has abnormalities related to leaf shape, leaf development and petiole development. The differentially expressed *CURLY LEAF* (*CLF*), *Growth-regulating factor 2* (*AtGRF2*), *AUXIN RESPONSE FACTOR 7* (*ARF7*), *BLADE ON PETIOLE2* (*BOP2*) and *KANADI 1*(*KAN1*) are promising candidate genes which possibly responsible for those abnormal leaf phenotypes (Table 2).

**Table 2:** Important DEGs and their candidate impact on the *flpl1* mutant

Upregulated gene ID	Gene name/common name	Impact on the mutant	References	Downregulated gene ID	Gene name/common name	Impact on mutant	References
AT2G39250	*SCHNARCHZAPFEN:SNZ*	Floral repressor	Mathieu et al. (2009)	AT2G45660	*AGAMOUS-LIKE 20:AGL20*	Floral promoter	Lee et al. (2000)
AT1G49130	*B-BOX DOMAIN PROTEIN 17:BBX17*	Floral repressor	Xu et al. (2022)	AT1G22770	*GIGANTEA: GI*	Floral promoter	Park et al. (1999)
AT3G24440	*VERNALIZATION 5:VRN5*	Floral repressor	Wood et al. (2006)	AT1G65480	*FLOWERING LOCUS T: FT*	Floral promoter	Kardailsky et al. (1999)
AT5G13790	*AGAMOUS-LIKE 15:AGL15*	Floral repressor	Adamczyk et al. (2007)	AT1G68050	*Flavin-binding, kelch repeat, f box 1: FKF1*	Floral promoter	Nelson et al. (2000)
AT5G03840	*TERMINAL FLOWER 1:TFL-1*	Floral repressor	Hanano and Goto (2011)	AT5G60100	*Pseudo-response regulator 3: APRR3;PRR3*	Floral promoter by stabilizes *TOC1* (*PRR1*)	Para et al. (2007)
AT2G26330	*ERECTA: ER: QRP1*	Petiole development	van Zanten et al. (2010)	AT5G60910	*Agamous-like 8: FRUITFULL:FUL*	Floral promoter	Ferrándiz et al. (2000)
AT1G04240	*IAA3;SHORT HYPOCOTYL 2;SHY2*	Leaf formation and root development	Tian and Reed (1999)	AT5G20730	*AUXIN RESPONSE FACTOR 7: ARF7*	Leaf and Petiole development	Wilmoth et al. (2005)
AT2G46970	*PHYTOCHROME INTERACTING FACTOR 3-LIKE 1;PIL1*	Petiole development	Lorrain et al. (2008)	AT3G18550	*BRANCHED 1: ATBRC1*	Bud and branch development, Petiole development	Aguilar-Martínez et al. (2007); González-Grandío et al. (2013)
AT4G37740	*growth-regulating factor 2: AtGRF2 GRF2*	Leaf shape and development	Kim et al. (2003)	AT2G41370	*BLADE ON PETIOLE2:BOP2*	Leaf and Petiole development	Ha et al. (2003)
AT2G23380	*CURLY LEAF:CLF*	Leaf shape and Floral repressor	Goodrich et al. (1997); Schubert et al. (2006)	AT5G16560	*KANADI 1: KAN1*	Leaf and Petiole development	Wu et al. (2008)

## Discussion

### Increased DNA content, an additional chromosome and the mutant genome

Based on the flow cytometry results, WGS coverage mapping analysis and the microscopic observation of the chromosomes, we were able to confirm that the *flpl1* plants possess an additional chromosome. The mutant cells are predicted to have 11 chromosomes, resulting in a *flpl1*/WT DNA content ratio of exactly 1.1 (Fig. 3b). However, we did not find any segregant with DNA ratios exceeding this value. When this mutant produces gametes by meiosis followed by self-fertilization, there can be five possible chromosome arrangements in the zygote. **1.** 5 (n) + 5 (n) – **10 (2n)**: WT type zygote. **2.** 5 (n) + 6 (n) – **10 (2n) + 1 (n)**: aneuploid heterozygous type zygote. **3.** 6 (n) + 6 (n) – **12 (2n)**: aneuploid homozygous type zygote. **4.** 5 (n) + 7 (n) – **10 (2n) + 2 (2n)**: aneuploid zygote (if one chromosome is lost due to misegregation or if an extra chromosome is retained due to nondisjunction). **5.** 6 (n) + 7 (n) – **10 (2n) + 3 (n)**: aneuploid zygote (due to misegregation or nondisjunction). The DNA ratio of 1.1 is assumed to match the above **2.** 5 (n) + 6 (n) – **10 (2n) + 1 (n)**: aneuploid heterozygous type of plant. If the plant is produced from the 3rd, 4th, or 5th types of zygotes, the DNA ratio should be ≥ 1.2. Since we could not find such a plant, the 3rd, 4th, or 5th type of zygote was assumed to be an embryonic lethal. Next, we calculated the expected increase in DNA content resulting from the additional Chr. 2, considering chromosome length and AT content (% of adenine (A) and thymine (T) bases), based on flow cytometry measurements. The results confirm that the *flpl1*/WT DNA content ratio of 1.1 is consistent with the addition of a single Chr. 2 to the *A. thaliana* genome (Tables S5, S6; Supplementary Text 2). Furthermore, we identified segmental aneuploid mutants that exhibited intermediate traits in terms of leaf shape, flowering time and DNA content between the wild type and the *flpl1* mutant (Fig. 5). These results suggest that there is a correlation between the increased DNA content, altered leaf shape and delayed flowering in these mutants (Fig. 5g).

The frequency of occurrence of *flpl1* mutants was continuous, yet at a slow rate, increasing in the consecutive inbred populations (Table S2). One of the possible reasons for this situation would be chromosomal instability, which disrupts normal chromosome pairing and segregation (non-disjunction) during meiosis (Henry et al. 2009; Huettel et al. 2008), leading to more 10 (2n) + 1 (n) offspring. If 10 (2n) + 1 (n) plants consistently produce gametes containing six chromosomes more frequently than expected, then crosses with gametes containing five chromosomes will continuously regenerate 10 (2n) + 1 (n) offspring at an increasing rate.

### The GO terms reveal insights into the *flpl1* phenotype

The GO terms represent an interesting overview of the whole transcriptome behavior of the mutant. Based on the BPs of the upregulated genes, the mutant cells are actively remodeling their cell wall structure, increasing cell division and growth through the mitotic cell cycle while ramping up protein production (via ribosomes). These biological processors create a relationship with GO terms of MF because the upregulated genes are involved in the functions of structural integrity, protein synthesis machinery, and cytoskeletal organization. The GO terms for CC further support the theme of enhanced protein synthesis capacity in the mutant because the highest number of upregulated genes is related to ribosomes. Collectively, the GO terms of upregulated genes correlate with the mutant phenotype characteristics of extended vegetative phase, larger leaves, and increased number of leaves because the mutant cells are assembling ribosomes, using them to synthesize structural proteins, and directing those proteins to active cell division, remodeling the cell wall and cytoskeleton. The additional chromosome in the mutant likely disrupts regulators that balance vegetative growth and flowering. Downregulation of plant organ senescence, leaf senescence, and response to decreased oxygen levels (hypoxia response) in the BP category of GO analysis is consistent with the *flpl1* phenotype. The mutant prioritizes vegetative growth by suppressing processes that limit growth or promote aging. Downregulation of the GO terms of MF related to salt and metal ion transmembrane transporter activity suggests that the mutant sacrifices stress resilience mechanisms for prioritization of growth because the downregulation of transmembrane related genes may reduce the capacity to manage salt stress or ion balance (Wu et al. 1996). Only three GO terms with low gene counts related to CC resulted in the analysis. Some of the downregulated DE genes may be functionally associated with cellular structures like root hairs and trichomes. Interestingly *flpl1* mutants possesses phenotypic abnormalities in the root and trichome structures (Figs. 1b, 2). Root hairs are plasma membrane-bounded projections extending from epidermal cells and trichomes are also cell projections that rely on cytoskeletal regulatory genes. Previous reports suggest that root hair mutants show defects in genes regulating cell projection growth (Galway et al. 1999; Yuen et al. 2005). Therefore, abnormal shallow root formation and trichome bases suggest defective cell projection formation, possibly due to downregulated CC terms. In summary, highly statistically significant up and downregulated GO terms are likely to correlate with mutant phenotypic features. As a whole, the mutant extends its vegetative phase by delaying flowering and increasing the biomass. The GO terms may expose the mechanisms that provide clues such as prioritizing the production of cell mass, structural components, and protein production while suppressing organ senescence.

### The phenotypic variations in the *flpl1* are driven by the additional copy of *A. thaliana* Chromosome 2

Classically, colchicine treatment has been used to obtain polyploid cells in *A. thaliana*. It disrupts the microtubule formation and longer exposures can inhibit entire cytokinesis (Henry et al. 2005; Parra-Nunez et al. 2020; Parra‐Nunez et al. 2024). In contrast, *flpl1* is a product of heavy-ion beam irradiation, which is a physical mutagenesis method. The mutant possesses a single additional chromosome, but two additional chromosome-containing plants were absent in the population. The WGS mutation analysis-based chromosome rearrangement suggested a fragmental translocation between Chr. 1 and Chr. 2. The paired ends of Chr.1 and Chr. 2 resulted as homozygous and heterozygous, respectively (mutation ID 1 and 2 in Fig. 4a and Table S3). In addition to this reciprocal translocation, a heterozygous ITX was found in Chr. 2. These results suggest that both copies of Chr. 1 have Chr. 2 fragments, two copies of Chr. 2 have Chr. 1 fragments due to CTX, and there should be a native copy of Chr. 2. Therefore, the sequence read coverage on Chr. 2 has increased by 50% (Fig. 4b). Supportively, *flpl1* resulted in a significantly higher amount of Chr. 2 DNA quantities compared to weak *flpl1* phenotype showing plants (Fig. S4). Microscopic observation also supports the presence of the additional chromosome (Fig. 3c). Therefore, we propose that the occurrence of the three copies of Chr. 2 is the most appropriate model (Fig. S2 ). In addition, previous results have shown that chromosomes appear in triplicate, and are vulnerable to breaking and rearranging (Huettel et al. 2008). The *flpl1*-77 mutant possesses such a rearranged additional Chr. 1. Even though we did not check the chromosomes’ structures, the intermediate DNA containing plants in Fig. 5 may retain such segmental aneuploid plants.

The previous study Henry et al. (2010) compared the phenotypic consequences of combinations of aneuploidy in *A. thaliana*. The aneuploidy mutant in Fig. 1c in Henry et al. (2010) shows a resemble phenotype with the *flpl1* mutant (Figs. 1, 5a) and its karyotype is 2n + 1 chromosome 2. This data supports the fact that the additional chromosome in the *flpl1* mutant may possess an extra copy of Chr. 2. Furthermore, the resemble phenotype supports the concept that similar phenotypes can be observed in individuals having the same karyotype (Henry et al. 2010). However, the impact on the phenotype varies from one individual to another, possibly due to external factors such as growth conditions. In the case of *flpl1*, since it’s an ion-beam-induced mutant line, additional mutations may also modify the phenotype. Mutations are prone to occur in the natural context, as a part of the evolutionary pathway. Even though aneuploidy or chromosome-level mutations have a low probability and are often disadvantageous in mutation breeding, they can induce significant phenotypic changes (Ikoma et al. 2025). As shown by the *flpl1* mutant, aneuploidy or chromosome-level mutations can be effective when multiple traits are desired, such as leaf shape, timing of vegetative to reproductive phase transition and root structures. High-LET heavy-ion beam irradiation, such as Ar and Fe ions, can cause large deletions, chromosomal rearrangements (Hirano et al. 2012, 2015; Kazama et al. 2017), and chromosomal fragmentation (Ishii et al. 2025), making it an effective mutagen for inducing mutations in multiple traits.

## Supplementary Information

Below is the link to the electronic supplementary material.

Supplementary file1 (PDF 1001 kb)

## Data Availability

Nucleotide sequence data files are available in the NCBI Sequenced Read Archive under the accession number SUB15363077 and SUB15611655 (https://www.ncbi.nlm.nih.gov/sra/PRJNA1271611) for *flpl1* (C30-81-as6) and *flpl1-*77 (C30-81-as6-77) respectively. The transcriptome data files are available in the NCBI Gene Expression Omnibus under the accession number GSE299045 (https://www.ncbi.nlm.nih.gov/geo/).
