# Supplementary material for: Arabidopsis aneuploidy-like mutant flpl1, possesses enormous variations in multiple phenotypic characteristics
Source: J Plant Res. 2026 May 13;139(4):629–44. doi: 10.1007/s10265-026-01712-5 (PMC13332973; doi:10.1007/s10265-026-01712-5)
Supplement: Supplementary file 1 — Supplementary file1 (PDF 1001 kb) [file 10265_2026_1712_MOESM1_ESM.pdf]

Title: Arabidopsis aneuploidy-like mutant *flpl1*, possesses enormous variations in multiple phenotypic characteristics.

Journal name: The Journal of Plant Research

Authors: Asanga Deshappriya Nagalla[1], Kotaro Ishii[1,2], Tomonari Hirano[1,3], Sumie Ohbu[1], Yuki Shirakawa[1], Yusuke Kazama[1,4], Tomoko Abe[1*]

The affiliations of the authors:

1. RIKEN Nishina Center, 2-1 Hirosawa, Wako, Saitama 351-0198, Japan

2. Department of Radiation Measurement and Dose Assessment, Institute for Radiological Science, Quantum Life and Medical Science Directorate, National Institutes for Quantum Science and Technology, Chiba 263-8555, Japan,

3. Faculty of Agriculture, University of Miyazaki, Miyazaki, Japan,

4. Department of Bioscience and Biotechnology, Fukui Prefectural University, Eiheiji-cho, Japan

* Corresponding author: tomoabe@riken.jp

Supplementary Text 1

Materials, methods and results for Linkage Analysis

We performed a linkage analysis using a BC_1F_2 population of 84 individuals and only one plant (*flpl1*-77) represented an intermediate phenotype of all three mutant characteristics: later flowering, crumpled large leaves and petioleless rosette leaves. The number of positive mutants was (the single intermediate plant) insufficient to perform linkage analysis. Therefore, we consider individuals showing at least two mutant characters. There were 22 plants showing later flowering, 21 plants showing crumpled, large leaves and one plant showing petioleless rosette leaves. Altogether, 13 plants both showed later flowering with crumpled large leaves and only one plant showed all three mutant characteristics (Fig. S5). We collected DNA from those 14 plants. The automated mutation analysis pipeline (AMAP; Ishii et al. 2016) resulted in nine homozygous mutated genes (Table S2) and was used for linkage analysis. However, we could not find a genetic linkage of the nine targets with at least a single mutant phenotype.

Method for quantitative PCR (qPCR) analysis

The genomic DNA was extracted from *flpl1* and *flpl1* weak mutants (the flowering period is similar to that of WT, the leaves contain the petiole, but the shape of the leaves is abnormal) and WT plants. All the plants were the same age. The *flpl1* and *flpl1* weak mutants were confirmed by the ploidy analyzer and phenotypic observation. DNA concentration was measured using a Qubit Fluorometer (Thermo Fisher Scientific, Waltham, MA, USA), diluted and the same quantities were used for qPCR analysis. The qPCR was performed using a THUNDERBIRDTM SYBR qPCR Mix (TOYOBO, Tokyo, Japan) on a CronoSTAR Real-Time PCR System (Takara Bio Inc., Shiga, Japan) according to the manufacturer's instructions. The control amplicon was on another chromosome (Chr. 5). Melt curve analysis results confirmed no secondary amplification for all the PCR products. Primers (I - VI) sequences were obtained from previously published (Ishii et al. 2024) and are listed in Table S7.

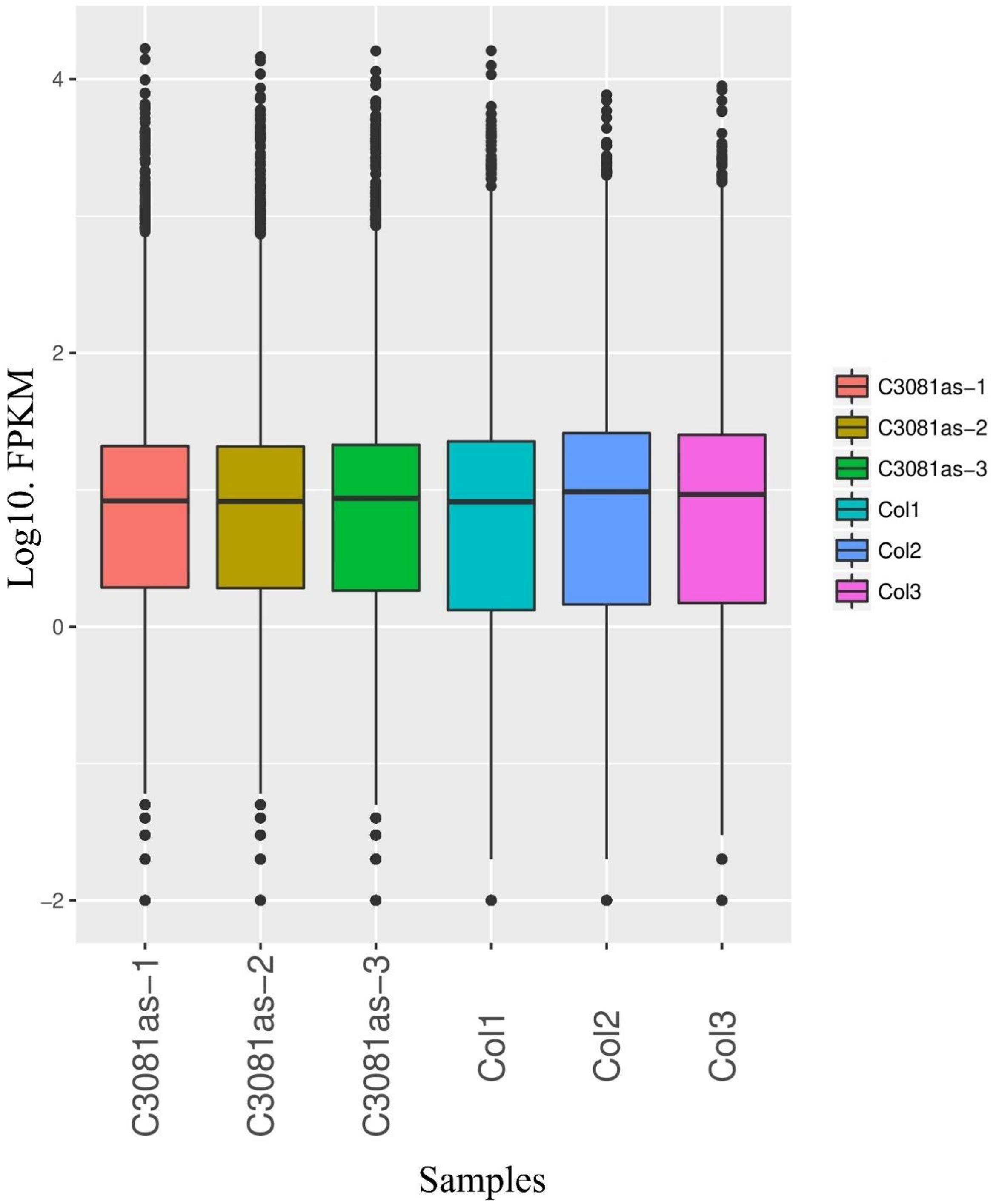

Fig. S1

Fragments per kilobase of transcript per million mapped reads (FPKM) plot box distribution of RNA-seq data visualize the distribution of gene expression levels across samples. C3081as stands for each *flpl1* sample and Col stands for WT.

a

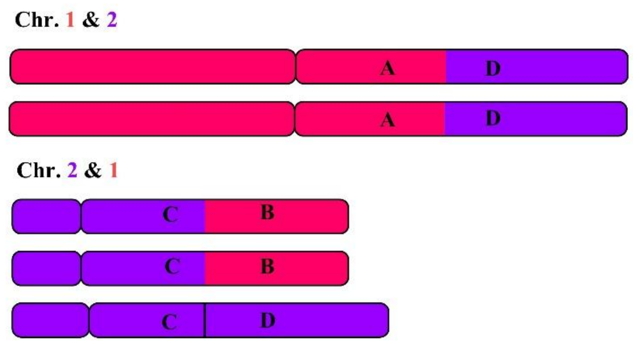

b

	Chr.1 Homo		Chr.2 Aneuploidy		
Fragment	A	D	D	C	B
Number of fragments	2	2	1	3	2

Fig. S2

Chromosome rearrangement model for *flpl1*. (**a**) Diagram of proposed reconstructed chromosome structures (Chr.1 and Chr. 2) of the *flpl1* mutant based on the automated mutation analysis pipeline and bam coverage mapping. The A, B, C and D stand for chromosome fragments. (**b**) Table summarizing the chromosomal fragments (A, B, C and D) in Chr. 1, Chr. 2 and their zygosity. Homo stands for homozygous and Chr. 2 remains aneuploid based on our model.

a

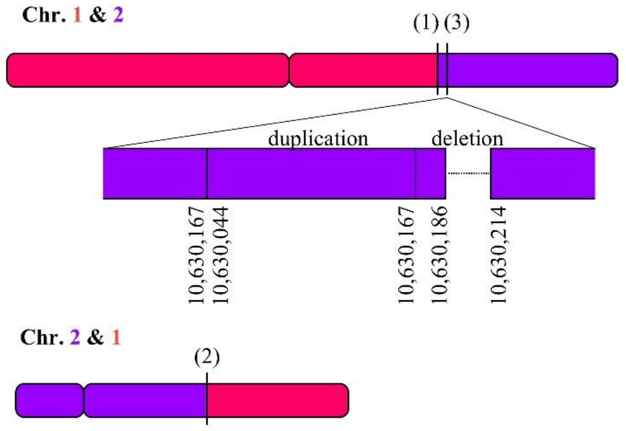

b

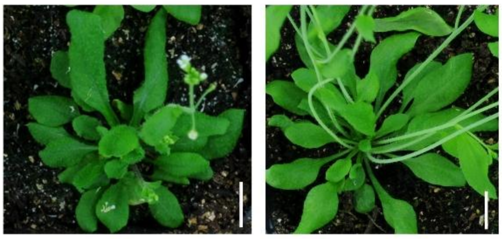

c

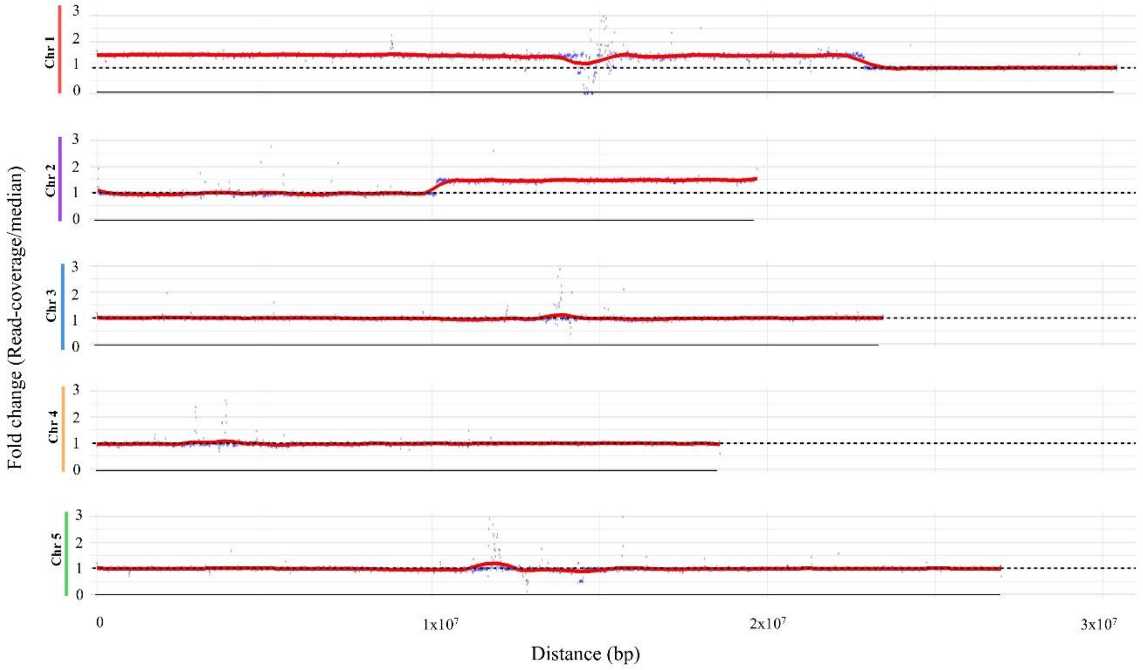

d

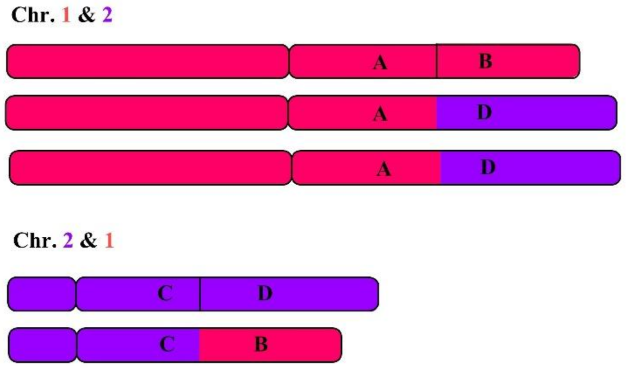

e

	Chr.1 Aneuploidy		Chr.2 Hetero	
**Fragment**	A	D	C	B
**Number of fragments**	3	3	2	2

Fig. S3

Visualization of additional chromosome fragments and phenotype of *flpl1*-77. (**a**) Diagram of proposed reconstructed chromosome structures of the *flpl1*-77 mutant based on the AMAP. The mutation IDs (1-3) represent the candidate break\rejoin locations as indicated in Table S3, based on TAIR 10. The sizes of the chromosome fragments are proportionate to the actual nucleotide sizes with respect to reference genome (Col-0) coordinates. (**b**) Phenotypic characteristics of the *flpl1*-77 (left) and the WT (right) plants. Photos were taken 49-days after germination. Bar = 1cm. (**c**)Whole genome sequence coverage plot for each chromosome. Coverage refers to the average reads per base in a 10kbp window indicated in blue dots (bin size =10kbp) and the median refers to the middle coverage value across the entire genome. The red color trend lines show the smoothed average coverage pattern across the genome and the black color dashed line refers to the base reference line. The x-axis shows positions along the chromosome in base pairs. (**d**) Diagram of proposed reconstructed chromosome structures (Chr.1 and Chr. 2) of the *flpl1*-77 mutant based on the automated mutation analysis pipeline and bam coverage mapping. The A, B, C and D stand for chromosome fragments. (**e**) Table summarizing the chromosomal fragments (A, B, C and D) in Chr. 1, Chr. 2 and their zygosity. Homo stands for homozygous and Chr. 1 remains aneuploid based on our model.

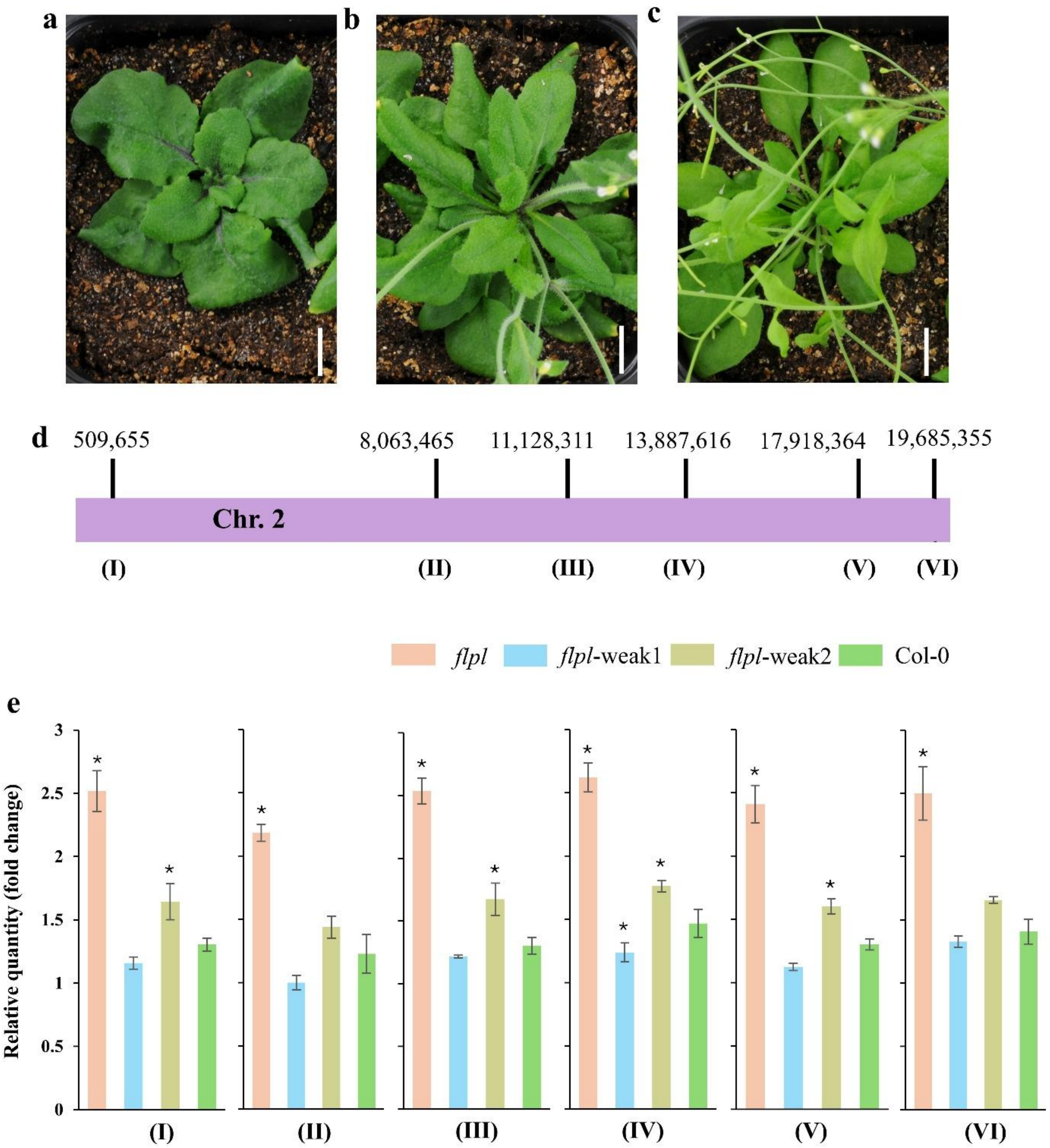

Fig. S4

The *flpl1* possesses an increased Chr. 2 quantity compared to *flpl1*-weak plants. (**a**) *flpl1*, (**b**) a representative *flpl1* weak (*flpl1*-weak) and (**c**) WT plants (photos were taken around 40 days after germination and Bars = 1cm). (**d**) Visualization of the positions of the quantitative PCR forward primer binding sites on Chr. 2. The size and the positions (in bp) of the chromosome fragment are proportional to the actual nucleotide sizes with respect to the reference genome (Col-0) coordinates. The primer sequences and position information are in Table S5. (**e**) The relative quantity of each amplicon (I - VI) in *flpl1*, two *flpl1*-weak mutants and WT. Bars

represent means ± standard deviation ($n = 3$ technical replicates). Statistical differences were determined by one-way ANOVA with Dunnett's test comparing each mutant to WT. (*$p < 0.05$).

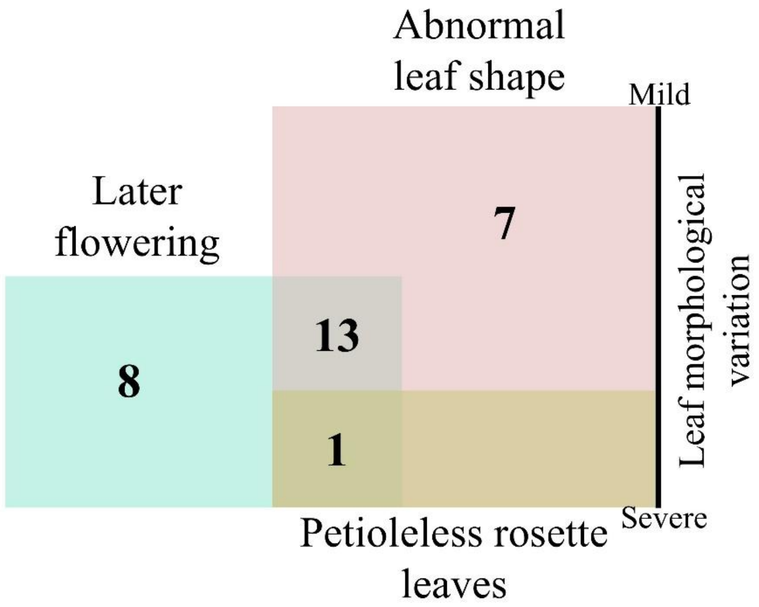

Fig. S5

The phenotypic characters and the number of plants observed in F_2 population.

Table S1: The trichome density between *flpl1* mutants and WT. An area of $2mm^2$ on the leaf blades was used for counting trichomes.

Position number	Total number of trichomes in *flpl1*	Total number of trichomes in WT
1	10	9
2	9	9
3	9	8
4	8	9
Trichome density	9 ± 0.71	8.75 ± 0.43

Table S2: The number of *flpl1* (positive) mutants (later flowering, crumpled, large and petioleless rosette leaves containing plants) in each generation.

Generation	Total number of plants	Number of *flpl1* mutants	% of *flpl1* mutants
M_3	30	1	3.3
M_4	33	3	9.0
M_5	40	10	25.0
M_7	19	7	36.8

Table S3: Whole genome sequence analysis results of *flpl1* mutant.

Chromosome 1	Position 1	Genetic homogenity	Chromosome 2	Position 2	Genetic homogenity	Reference	Mutated base	Type of mutation	Effect type for gene	Gene ID affected by position 1	Gene ID affected by position 2	Mutation ID
**1**	1,279,595	**HOM**				C	A	SNP	missense_variant	AT1G04610		
**1**	3,150,930	**HOM**				G	A	SNP	synonymous_variant	AT1G09730		
**1**	7,285,221	HET				C	A	SNP				
**1**	10,085,618	HOM				G	A	SNP				
**1**	11,639,575	HOM				AT	A	DEL				
**1**	17,740,018	HOM				T	C	SNP				
**1**	18,557,695	HOM				A	G	SNP				
**1**	20,690,572	HOM				T	A	SNP				

**1**	22,888,469	**HOM**	2	10,120,869	**HET**			CTX	trancation		AT2G23770	**(1)**
**1**	22,888,477	**HOM**	2	10,120,491	**HET**			CTX	trancation		AT2G23770	**(2)**
**1**	26,521,774	HOM				AT	A	DEL				
**1**	28,293,670	HOM				GA	G	DEL				
**2**	653,478	HET				T	A	SNP	missense_variant	AT2G02470		
**2**	653,480	HET				C	A	SNP	missense_variant	AT2G02470		
**2**	9,992,666	HET				TTG	T	DEL				
**2**	10,630,044	HET	2	10,630,167	**HET**			ITX				**(3)**
**2**	10,630,186	HET					27 bp DEL	DEL				
**2**	10,958,868	HET				A	C	SNP				
**2**	11,263,038	HET				T	A	SNP				

**2**	14,154,316	HET				A	T	SNP	splice_donor_variant&intron_variant	AT2G33400		
**2**	16,930,529	HOM				AC	A	DEL				
**2**	19,205,018	HOM				AT	A	DEL				
**3**	1,709,369	**HOM**				C	T	SNP	stop_gained	AT3G05760		
**3**	5,528,102	**HOM**				C	T	SNP	missense_variant	AT3G16310		
**3**	7,581,255	HOM				C	T	SNP				
**3**	11,719,665	HET				C	A	SNP				
**3**	11,738,017	HET				C	CA	INS				
**3**	14,315,102	HET				C	T	SNP				
**3**	14,393,600	HET	3	16,127,754	HET			ITX	trancation		AT3G44540	**(4)**
**3**	14,393,875	HET	3	16,127,768	HET			ITX	trancation		AT3G44540	**(5)**

**3**	14,711,314	HET				A	G	SNP			
**3**	16,235,901	HET				TTC	T	DEL			
**3**	18,217,794	HET				A	T	SNP	missense_variant	AT3G49142	
**3**	19,710,233	HET				G	C	SNP	splice_region_variant&intron_variant	AT3G53180	
**3**	19,879,576	HET				G	T	SNP			
**3**	22,702,225	**HOM**				A	C	SNP	missense_variant	AT3G61340	
**3**	23,052,522	HOM				A	T	SNP			
**3**	23,360,253	**HOM**					138 bp DEL	DEL	frameshift_variant	AT3G63230	
**4**	347,586	HOM				T	C	SNP			
**4**	2,913,481	HOM					30 bp DEL	DEL			
**4**	6,153,345	HET	4	9,111,121	HET			ITX			**(6)**

**4**	6,153,351	HET	4	9,111,129	HET			ITX			**(7)**
**4**	8,928,591	HET				G	A	SNP			
**4**	9,298,550	HET				G	C	SNP			
**4**	10,709,765	HET				C	A	SNP			
**4**	11,911,295	HOM				A	G	SNP			
**4**	12,724,082	**HOM**	3	(repetitive region)				CTX	trancation	AT4G24660	**(8)**
**4**	12,724,136	**HOM**	4	16,169,009	**HET**			CTX	trancation	AT4G24660	**(9)**
**4**	16,169,005	HET	4	16,242,056	HET			CTX			**(10)**
**4**	16,241,835	HET	3	(repetitive region)				CTX			**(11)**
**4**	15,826,641	HET				G	T	SNP			

**4**	15,826,642	HET			A	T	SNP			
**4**	16,359,953	HET			GA	G	DEL			
**5**	613,920	HET			C	A	SNP	synonymous_variant	AT5G02720	
**5**	613,921	HET			G	GTTGTACACAAA	INS	frameshift_variant	AT5G02720	
**5**	10,528,505	**HOM**	5	(repetitive region)			ITX			**(12)**
**5**	10,528,701	**HOM**	5	(repetitive region)			ITX			**(13)**
**5**	16,740,452	HET			A	C	SNP			
**5**	16,867,074	HET			C	CGG	INS	frameshift_variant	AT5G42210	
**5**	21,853,363	HOM			CA	C	DEL			
**5**	23,199,299	**HOM**			C	A	SNP	stop_gained	AT5G57260	

**5**	26,711,631	HOM		CCTATGGA	C	DEL

HOM: homozygous, HET: heterozygous, SNP: single nucleotide polymorphism, DEL: deletion, INS: insertion, ITX: intra-chromosomal translocation, CTX: inter-chromosomal translocation

Table S4: Whole genome sequence analysis results of *flpl1*-77 mutant.

Chromosome 1	Position 1	Genetic homogenity	Chromosome 2	Position 2	Genetic homogenity	Reference	Mutated base	Type of mutation	Effect type for gene	Gene ID affected by position 1	Gene ID affected by position 2	Mutation ID
**1**	8,764	HET				C	A	SNP				
**1**	3,093,370	HET				C	T	SNP				
**1**	3,190,735	HET				T	TA	INS	frameshift_variant	AT1G09820		
**1**	3,190,736	HET				G	C	SNP	missense_variant	AT1G09820		
**1**	7,925,164	HET				TA	T	DEL				
**1**	1,008,5618	HET				G	A	SNP				
**1**	1,1639,575	HET				AT	A	DEL				
**1**	17,740,018	HET				T	C	SNP				
**1**	18,341,659	HET				T	G	SNP				

**1**	18,557,695	HET				A	G	SNP			
**1**	20,690,572	HET				T	A	SNP			
**1**	22,888,469	**HET**	2	10,120,869	**HET**			CTX	trancation	AT2G23770	**(1)**
**1**	22,888,477	**HET**	2	10,120,491	**HET**			CTX	trancation	AT2G23770	**(2)**
**2**	8,682,241	HET				G	A	SNP			
**2**	10,630,044	**HET**	2	10,630,167	**HET**			ITX			**(3)**
**2**	10,630,186	HET					27 bp DEL	DEL			
**2**	11,263,038	HET				T	A	SNP			
**2**	14,123,848	HET				T	G	SNP			
**2**	16,930,529	HET				AC	A	DEL			
**2**	19,205,018	HET				AT	A	DEL			

**3**	1,217,<br>043	HOM	G	C	SNP		
**3**	1,709,<br>36,9	HOM	C	T	SNP	stop_gained	AT3G05<br>760
**3**	55,28<br>102	HOM	C	T	SNP	missense_variant	AT3G16<br>310
**3**	7,581,<br>255	HOM	C	T	SNP		
**3**	11,71<br>9,665	HOM	C	A	SNP		
**3**	13,72<br>3,518	HOM	AGTTTGGCCTCATG<br>GTCTC	A	DEL		
**3**	13,72<br>3,540	HOM	G	A	SNP		
**3**	13,72<br>3,541	HOM	T	A	SNP		
**3**	13,72<br>3,542	HOM	T	A	SNP		
**3**	14,31<br>5,102	HOM	C	T	SNP		
**3**	15,18<br>6,549	HOM	GGTCAAAATTTTTTA<br>TTGCCATACCAT	G	DEL		

**3**	15,186,550	HOM		27 bp DEL	DEL		
**3**	16,235,901	HOM	TTC	T	DEL		
**3**	18,217,794	HOM	A	T	SNP	missense_variant	AT3G49142
**3**	19,710,233	HOM	G	C	SNP		
**3**	22,702,225	HOM	A	C	SNP	missense_variant	AT3G61340
**4**	347,586	HOM	T	C	SNP		
**4**	2,913,480	HOM		30 bp DEL	DEL		
**4**	8,928,591	HET	G	A	SNP		
**4**	9,298,550	HET	G	C	SNP		
**4**	10,709,765	HET	C	A	SNP		
**4**	16,359,953	HET	GA	G	DEL		

**4**	1,750,9276	HET	G	T	SNP	missense_variant	AT4G37190
**5**	8,489,292	HOM	T	C	SNP	missense_variant	AT5G24740
**5**	23,199,299	HET	C	A	SNP	stop_gained	AT5G57260
**5**	26,711,631	HET	CCTATGGA	C	DEL		

HOM: homozygous, HET: heterozygous, SNP: single nucleotide polymorphism, DEL: deletion, INS: insertion, ITX: intra-chromosomal translocation, CTX: inter-chromosomal translocation

Table S5: DNA quantity of each peak measured by the ploidy analyzer.

Plant	2C	4C	8C	16C
Col-0	**4,751 ± 119**	9,078.6 ± 156.3	17,744.2 ± 248.6	34,866.3 ± 477.5
flpl1	**5,186.6 ± 25.7 ***	10,144.9 ± 116.7 *	19,877.3 ± 321.4 *	39,109.3 ± 672.6 *
flpl1/Col-0	**1.09**	1.12	1.12	1.12

Data are means ± SD ($n \geq 3$). * The significance of the difference was assessed by Student's t-test (*$p < 0.01$).

Table S6: *A. thaliana* chromosome length and AT %

Chromosome	Length (Mb)	AT%
1	30.4	64.13
2	19.7	64.14
3	23.5	63.67
4	18.6	63.8
5	27.0	64.07

The chromosome length and AT% were calculated based on the *A. thaliana* reference genome TAIR10.1

Supplementary Text 2

Assumption: Effective DAPI signal ∝ Length × AT fraction

Approximate Chromosome contribution for DAPI signal:

Chr1: 30.4×0.641264 ≈ 19.50

Chr2: 19.7×0.641357 ≈ 12.63

Chr3: 23.5×0.636689 ≈ 14.96

Chr4: 18.6×0.637960 ≈ 11.87

Chr5: 27.0×0.640611 ≈ 17.30

Total AT-weighted 1C : ≈ 76.26

Total AT-weighted 2C : ≈ 152.52

Calculation of the increment of DNA quantity due to extra Chr. 2

Add extra Chr2 (AT-weighted): 165.15 (152.52 + 12.63)

Relative increase: 12.63/152.52 = 0.0828 (≈8.28%)

Approximate DNA Ratio: 165.15/152.52 =**1.0828**

Scale the DAPI 2C peak: 4,751×1.0828 = **5,145**

The calculated additional DNA quantity in *flpl1* is approximately comparable to the estimated DNA quantity using Flow cytometry.

Table S7: Primers used for quantitative PCR analysis. The primer numbers are the same as the numbers that appeared in Fig. S4.

Primer number	Forward primer sequence (5'-3')	Forward primer position	Reverse primer sequence (5'-3')	Reverse primer position
I	tttttggctgacacatttatcg	2: 509,655	aaggctgcttgtactctccaa	2: 509,726
II	tggcttgggtcacctcat	2: 8,063,465	tttaggacggaggagagacg	2: 8,063,524
III	cgttttgctaccaatgtgga	2: 11,128,311	agatcatctctgccaagacca	2: 11,128,374
IV	catccaaagagccgaacc	2: 13,887,616	cgtctctgcatcatcgtcac	2: 13,887,677
V	aaacccaccaaaggagacg	2: 17,918,364	agagagagagattgaagaaacaaacc	2: 17,918,427
VI	ccaacggagggaactgtct	2: 19,685,355	tctccttgcacatcggtaca	2: 19,685,414
Control	aatcatttcttcttcgattccttctatttc	5: 24,916,469	gacgtaattcgacatgagactatagatatt	5: 24,916,896
